# The Risk of Nephropathy, Retinopathy, and Leg Amputation in Patients With Diabetes and Hypertension: A Nationwide, Population-Based Retrospective Cohort Study

**DOI:** 10.3389/fendo.2021.756189

**Published:** 2021-11-18

**Authors:** Fu-Shun Yen, James Cheng-Chung Wei, Ying-Hsiu Shih, Chih-Cheng Hsu, Chii-Min Hwu

**Affiliations:** ^1^ Dr. Yen’s Clinic, Taoyuan, Taiwan; ^2^ Department of Allergy, Immunology & Rheumatology, Chung Shan Medical University Hospital, Taichung City, Taiwan; ^3^ Institute of Medicine, Chung Shan Medical University, Taichung City, Taiwan; ^4^ Graduate Institute of Integrated Medicine, China Medical University, Taichung City, Taiwan; ^5^ Management Office for Health Data, China Medical University Hospital, Taichung, Taiwan; ^6^ College of Medicine, China Medical University, Taichung City, Taiwan; ^7^ Institute of Population Health Sciences, National Health Research Institutes, Zhunan, Taiwan; ^8^ Department of Health Services Administration, China Medical University, Taichung, Taiwan; ^9^ Department of Family Medicine, Min-Sheng General Hospital, Taoyuan, Taiwan; ^10^ Faculty of Medicine, National Yang-Ming University School of Medicine, Taipei, Taiwan; ^11^ Section of Endocrinology and Metabolism, Department of Medicine, Taipei Veterans General Hospital, Taipei, Taiwan

**Keywords:** chronic kidney disease, end-stage renal disease, sight-threatening retinopathy, leg amputation, diabetes and hypertension

## Abstract

**Purpose:**

To compare the risks of chronic kidney disease (CKD), end-stage renal disease (ESRD), sight-threatening retinopathy, and leg amputation between patients with diabetes or hypertension.

**Methods:**

From January 1, 2000, to December 31, 2015, we identified 28943 matched pairs of patients with diabetes with and without subsequent hypertension, 89102 pairs of patients with hypertension with and without subsequent diabetes, and 145294 pairs of patients with coexisting diabetes and hypertension with a previous history of diabetes or hypertension from Taiwan’s National Health Insurance Research Database. Cox proportional-hazard models were used for calculating the risks of CKD, sight-threatening retinopathy, and leg amputation.

**Results:**

The mean follow-up time of this study in different cohorts was between 3.59 and 4.28 years. In diabetes patients with vs. without subsequent hypertension, hypertension patients with vs. without subsequent diabetes, and comorbid diabetes and hypertension patients with previous diabetes vs. with previous hypertension, the adjusted HRs (95% CIs) for CKD were 2.77 (2.61-2.94), 1.73 (1.68-1.77), and 1.04 (1.02-1.07); for ESRD were 42.38 (22.62-79.4), 2.76 (2.43-3.13), and 0.72 (0.66-0.79); for sight-threatening retinopathy were 2.07 (1.85-2.3), 3.41 (3.14-3.71), and for leg amputation were 1.51 (1.43-1.58); and 4.74 (3.02-7.43), 6.27(4.72-8.31), and 1.19(1.03-1.38).

**Conclusions:**

This study demonstrated that both diabetes and hypertension are risk factors for the development of CKD, retinopathy, and amputation. Tracing subsequent diabetes for patients with hypertension, and hypertension for patients with diabetes are important in clinical settings.

## Introduction

Hypertension is one of the most common chronic diseases in the world (1). It can lead to cardiovascular diseases and chronic kidney disease (2). High systolic blood pressure is the leading risk factor for attributable deaths, accounting for 10.8 million deaths worldwide and 19.2% of all deaths in 2019 (3). Type 2 diabetes mellitus, one of the leading chronic diseases globally, is linked to lifestyle factors. In 1990, approximately 148 million people worldwide had diabetes, and the number tripled to about 438 million in 2019. The prevalence rate also increased from 2.88% in 1990 to 5.89% in 2019 (4). Patients with diabetes are prone to developing macrovascular and microvascular complications, which increase the risk of mortality.

Chronic kidney disease is a silent deterioration of renal function to estimated glomerular filtration rate (eGFR) less than 60 ml/min/1.73m^2^ or abnormal markers of renal damage for more than 3 months (5). If unmanaged, it may progress to end-stage renal disease (ESRD) and increase the risk of cardiovascular disease and premature death (5). Retinopathy involves abnormal changes in the small retinal blood vessels. It is the leading cause of blindness among working-age adults (6). Poor blood circulation in the distal limbs can lead to leg amputation, the last resort in managing poorly healing wounds that worsens the quality of life in patients (7).

Reports suggest that hypertension and diabetes are the main risk factors for CKD, retinopathy, and amputation (5, 8, 9). Diabetes is the most common cause of ESRD; up to 80% of ESRD is caused by diabetes, hypertension, or a combination of both (10). Population aging in Taiwan has resulted in an increasing prevalence of diabetes and CKD in recent years. Taiwan has the highest incidence and prevalence of dialysis in the world (11). Because few studies have investigated the different impacts of diabetes and hypertension on microvascular complications, we conducted this study to compare whether diabetes with or without subsequent hypertension, hypertension with or without subsequent diabetes, and coexisting hypertension and diabetes with a previous history of hypertension or diabetes, differ in their impacts on the risks of CKD, ESRD, sight-threatening retinopathy, and leg amputation.

## Materials and Methods

### Study Population

We identified patients with newly diagnosed type 2 diabetes mellitus or hypertension from the National Health Insurance Research Database (NHIRD) between January 1, 2000, and December 31, 2015. The NHIRD contains medical records of National Health Insurance (NHI) from 1995 to the present (12). It includes information on patient sex, age, place of residence, procedure, therapy, and diagnosis according to the International Classification of Diseases, Ninth Revision, Clinical Modification (ICD-9-CM), and ICD-10-CM codes. It involves the health services of inpatient admissions, outpatient visits, and emergency department visits. The NHIRD is linked to the National Death Registry to certify mortality information. The NHI program was implemented by the Taiwan government in 1995. It is a compulsory insurance system, with most of the premiums paid by the government and employers. By 2000, approximately 99% of the 23 million people in Taiwan were insured. Our study was approved by the Research Ethics Committee of China Medical University and Hospital (CMUH109-109-REC2-031). The identifier information of patients or care providers was di-identified and encrypted before release to protect individual privacy. Therefore, informed consent from patients was not required.

### Study Design

Type 2 diabetes mellitus and hypertension were diagnosed by ICD-9-CM codes or ICD-10-CM codes (**Supplementary Table 1**), with at least 2 outpatient claims within 1 year or one hospitalization. This algorithm of using ICD codes has been validated by previous studies with the accuracy of diabetes was 74.6% (13), the sensitivity and specificity of hypertension were 92.4% and 59.9% (14). We excluded patients diagnosed with type 1 diabetes, younger than 20 years or older than 80 years (patients with too old age could have poor renal function or frail syndromes which may interfere with the results), lacking age or gender information, diagnosed with chronic kidney disease (CKD), having dialysis, retinopathy, visual loss, leg amputation, heart failure, and hepatic failure before the index date. We also excluded patients who died or were followed-up for less than 180 days after the index date (to avoid confounding effects of the latent morbidities).

### Procedures

**Figure 1:**
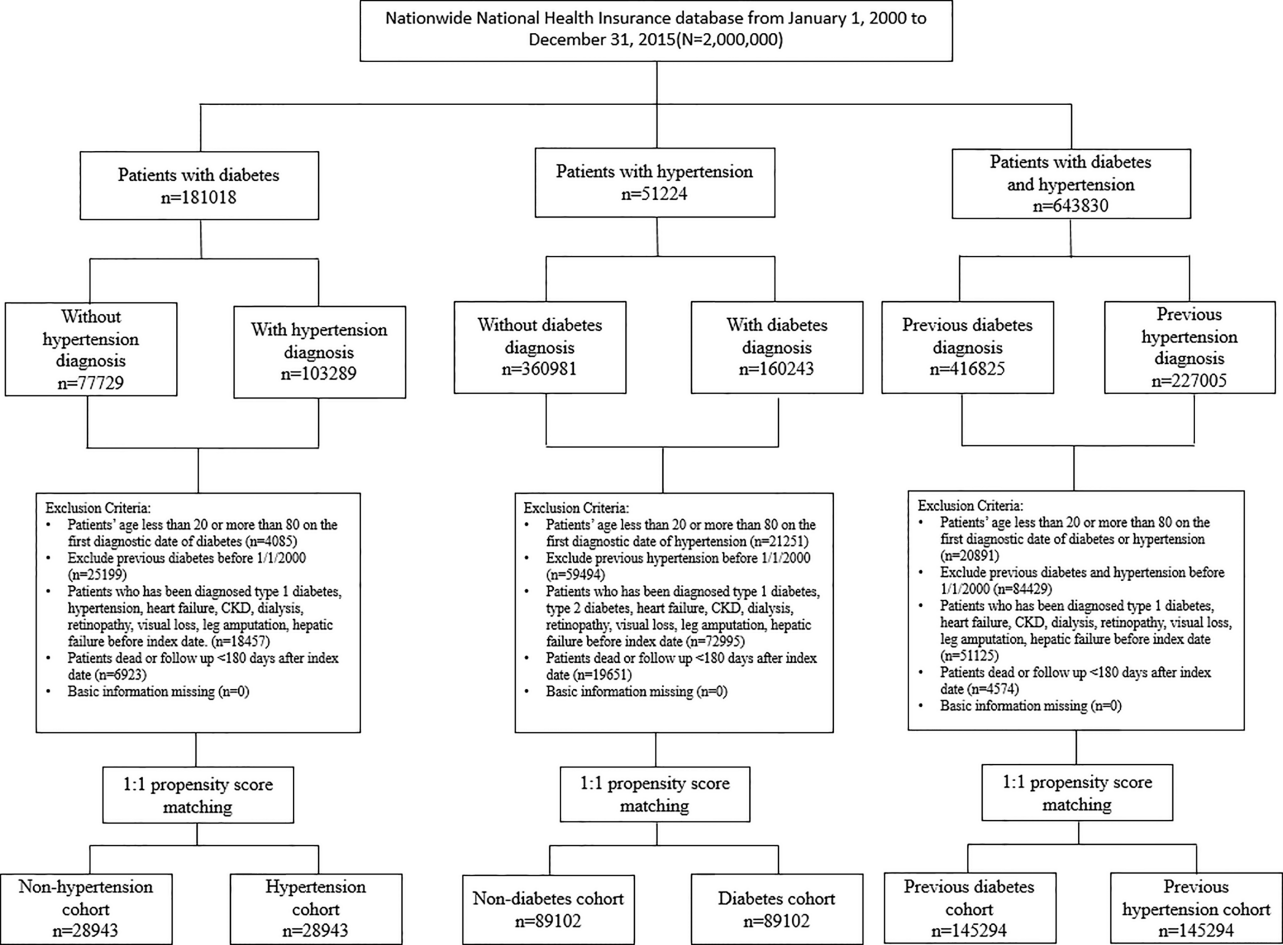
Flow chart of the identified study population.

In this retrospective cohort study, we constructed 3 cohorts from 2000 to 2015 to compare the risks of CKD, retinopathy, and amputation in relation to diabetes and hypertension (**Figure 1**): (a) diabetes patients with and without subsequent hypertension (diabetes cohort), (b) hypertension patients with and without subsequent diabetes (hypertension cohort), (c) patients with coexisting diabetes and hypertension (comorbid cohort). The coexisting diabetes and hypertension indicates a patient has at least 2 outpatient claims within 1 year or one hospitalization due to both diseases. For the diabetes cohort, of 181018 newly diagnosed diabetes patients, after excluding ineligible patients, there were 103289 patients with subsequent hypertension and 77729 patients without subsequent hypertension. We defined the date of first hypertension diagnosis as the index date of this diabetes cohort. For the hypertension cohort, of 51224 newly diagnosed hypertension patients, after excluding ineligible patients, there were 160243 patients with subsequent diabetes and 360981 patients without subsequent diabetes. We defined the date of subsequent diabetes diagnosis as the index date of this hypertension cohort. For the comorbid cohort, of 643830 patients with coexisting diabetes and hypertension, after excluding ineligible patients, there were 416825 patients with previous diabetes and 227005 patients with previous hypertension. We defined the date of concurrent diabetes and hypertension diagnosis as the index date of this comorbid cohort. Within each cohort, we assigned the same index date for the two comparison subgroups to avoid immortal time bias. The detailed study designs were delineated in **Supplementary Table 2**.

Variables considered as potential confounders in this study were as follows: sex, age, overweight, obesity, severe obesity, smoking, dyslipidemia, coronary artery disease (CAD), stroke, atrial fibrillation, chronic obstructive pulmonary disease (COPD), liver cirrhosis, peripheral arterial occlusion disease (PAOD); Charlson Comorbidity Index (CCI) (15) and Diabetes Complication Severity Index (DCSI) scores (16); number and item of oral antidiabetic medications and insulin (**Table 1**); number and item of antihypertensive medications (**Table 2**); non-steroidal anti-inflammatory drugs (NSAIDs); statin; aspirin (**Tables 2**, **3**); duration of diabetes (the duration of first diabetes diagnosis to the index date. **Table 1**); duration of hypertension (the duration of first hypertension diagnosis to the index date. **Table 2**).

**Table 1:** Comparison of baseline characteristics of the study subjects in the diabetes cohort.

Variables	Without subsequent hypertension		With subsequent hypertension		SMD
	(N = 28943)		(N = 28943)		
	n	%	n	%	
Sex					
Female	13464	46.52	13454	46.48	0.001
Male	15479	53.48	15489	53.52	0.001
Age					
20-39	3920	13.54	3939	13.61	0.002
40-59	17477	60.38	17450	60.29	0.002
60-80	7546	26.07	7554	26.10	0.001
Mean, (SD)	53.2	11.48	53.21	11.51	0.001
Comorbidities					
Obesity					
Overweight	560	1.93	564	1.95	0.001
Normal Obesity	451	1.56	441	1.52	0.003
Severe obesity	53	0.18	59	0.20	0.005
Smoking	588	2.03	618	2.14	0.007
Dyslipidemia	15816	54.65	16107	55.65	0.02
Coronary artery disease	3897	13.46	3872	13.38	0.003
Stroke	1197	4.14	1152	3.98	0.008
Atrial fibrillation	11	0.04	16	0.06	0.008
PAOD	656	2.27	671	2.32	0.003
COPD	5643	19.50	5766	19.92	0.011
Liver cirrhosis	744	2.57	772	2.67	0.006
CCI					
1	9540	32.96	9289	32.09	0.019
2-3	13933	48.14	13981	48.31	0.003
>3	5470	18.90	5673	19.60	0.018
DCSI					
0	13902	48.03	13863	47.90	0.003
1	5809	20.07	5803	20.05	0.001
≥2	9232	31.90	9277	32.05	0.003
Medication					
Metformin	13849	47.85	13957	48.22	0.007
Sulfonylurea	12719	43.95	13323	46.03	0.042
TZD	2680	9.26	2774	9.58	0.011
DPP-4i	1571	5.43	1409	4.87	0.025
AGI	2718	9.39	2918	10.08	0.023
Number of OAD					
0-1	17424	60.20	17267	59.66	0.011
2-3	10046	34.71	10184	35.19	0.01
>3	1473	5.09	1492	5.16	0.003
Insulin	10369	35.83	10377	35.85	0.001
Statin	8082	27.92	8270	28.57	0.02
NSAIDs	28139	97.22	28231	97.54	0.014
Diabetes duration, (SD)	3.69	3.33	3.59	3.51	0.03

SMD, standardized mean difference. A standardized mean difference of 0.05 or less indicates a negligible difference.

PAOD, peripheral arterial occlusive disease; COPD, chronic obstructive pulmonary disease; CCI, Charlson comorbidity index; DCSI, diabetes complication severity index; TZD, thiazolidinedione; DPP-4i, Dipeptidyl peptidase-4 inhibitor; AGI, Alpha-glucosidase inhibitors; NSAIDs, non-steroidal anti-inflammatory drugs; OAD, oral anti-diabetic drugs.

**Table 2:** Comparison of baseline characteristics of the study subjects in the hypertension cohort.

Variables	Without subsequent diabetes		With subsequent diabetes		SMD
	(N = 89102)		(N = 89102)		
	n	%	n	%	
Sex					
Female	43646	48.98	44382	49.8	0.017
Male	45456	51.02	44720	50.2	0.017
Age					
20-39	5613	6.30	5604	6.3	0
40-59	44125	49.52	44279	49.7	0.003
60-80	39364	44.18	39219	44.0	0.003
mean, (SD)	58.58	11.50	58.55	11.5	0.003
Obesity					
Overweight	1055	1.18	1174	1.3	0.012
Normal Obesity	831	0.93	917	1.0	0.01
Severe obesity	122	0.14	160	0.2	0.011
Smoking status	1074	1.21	1274	1.4	0.02
Comorbidities					
Dyslipidemia	36784	41.28	38830	43.6	0.046
Coronary artery disease	24956	28.01	26648	29.9	0.042
Stroke	20	0.02	13	0.0	0.006
Atrial fibrillation					
PAOD	2217	2.49	2569	2.9	0.024
COPD	21069	23.65	22328	25.1	0.033
Liver cirrhosis	1147	1.29	1402	1.6	0.024
CCI					
1	28099	31.54	26612	29.9	0.036
2-3	43709	49.06	43636	49.0	0.002
>3	17294	19.41	18854	21.2	0.044
Medication					
ACEI/ARB	30382	34.10	31026	34.8	0.015
β-blockers	59179	66.42	59203	66.4	0.001
Calcium-channel blockers	64955	72.90	64742	72.7	0.005
Diuretics	43497	48.82	46378	52.1	0.065
Number of hypertension drugs					
1	26787	30.06	25079	28.1	0.042
2-3	46939	52.68	47825	53.7	0.02
>3	15376	17.26	16198	18.2	0.024
Statin	19498	21.88	20877	23.4	0.037
Aspirin	32775	36.78	34187	38.4	0.033
NSAIDs	84529	94.87	85543	96.0	0.055
Hypertension duration, (SD)	4.28	3.58	4.26	3.69	0.005

SMD, standardized mean difference. A standardized mean difference of 0.05 or less indicates a negligible difference.

PAOD, peripheral arterial occlusive disease; COPD, chronic obstructive pulmonary disease; CCI, Charlson comorbidity index; ACEI, angiotensin converting enzyme inhibitors; ARB, angiotensin receptor blockers; NSAIDs, non-steroidal anti-inflammatory drugs.

**Table 3:** Comparison of baseline characteristics of the study subjects in the comorbid cohort.

Variables	With previous diabetes		With previous hypertension		SMD
	(N = 145294)		(N = 145294)		
	n	%	n	%	
Sex					
Female	72020	49.57	72937	50.20	0.013
Male	73274	50.43	72357	49.80	0.013
Age					
20-39	20128	13.85	18948	13.04	0.024
40-59	76192	52.44	75399	51.89	0.011
60-80	48974	33.71	50947	35.06	0.029
Mean, (SD)	54.69	12.73	55.18	12.75	0.038
Obesity					
Overweight	1486	1.02	1454	1.00	0.002
Obesity	1101	0.76	1097	0.76	0
Severe obesity	150	0.10	199	0.14	0.01
Smoking status	1872	1.29	1869	1.29	0
Comorbidities					
Dyslipidemia	51072	35.15	50562	34.80	0.007
Coronary artery disease	27611	19.00	28518	19.63	0.016
Stroke	8022	5.52	8451	5.82	0.013
Atrial fibrillation	971	0.67	1012	0.70	0.003
PAOD	2669	1.84	2841	1.96	0.009
COPD	29757	20.48	29649	20.41	0.002
Liver cirrhosis	2341	1.61	2224	1.53	0.006
CCI					
0	52737	36.30	53426	36.77	0.01
1	67119	46.20	65933	45.38	0.016
≥2	25438	17.51	25935	17.85	0.009
DCSI					
0	80797	55.61	81112	55.83	0.009
1	25688	17.68	26997	18.58	0.009
≥2	38809	26.71	37185	25.59	0.009
Medications					
Metformin	22843	15.72	16833	11.59	0.121
Sulfonylurea	23979	16.50	18466	12.71	0.108
TZD	4934	3.40	1406	0.97	0.167
DPP-4i	1435	0.99	424	0.29	0.087
AGI	4727	3.25	1546	1.06	0.151
Insulin	39909	27.47	37694	25.94	0.034
Number of OAD					
≦1	125118	86.11	133454	91.85	0.009
2-3	17631	12.13	11548	7.95	0.009
>3	2545	1.75	292	0.20	0.009
ACEI/ARB	9103	6.27	34867	24.00	0.511
β-blockers	47858	32.94	73983	50.92	0.371
Calcium-channel blockers	30195	20.78	73095	50.31	0.648
Diuretics	31638	21.78	55676	38.32	0.367
Number of hypertension drugs					
1	113187	77.90	72356	49.80	0.612
2-3	30476	20.98	56009	38.55	0.392
>3	1631	1.12	16929	11.65	0.441
Statin	22774	15.67	23502	16.18	0.014
Aspirin	37910	26.09	38930	26.79	0.016
NSAIDs	134184	92.35	133289	91.74	0.023
Diabetes duration					1.601
mean, (SD)	3.79	3.35	–	–	
Hypertension duration					1.621
mean, (SD)	–	–	4.07	3.55	

SMD, standardized mean difference. A standardized mean difference of 0.05 or less indicates a negligible difference.

PAOD, peripheral arterial occlusive disease; COPD, chronic obstructive pulmonary disease; CCI, Charlson comorbidity index; DCSI, Diabetes Complication Severity Index; TZD, thiazolidinedione; DPP-4i, Dipeptidyl peptidase-4 inhibitor; AGI, Alpha-glucosidase inhibitors; ACEI, angiotensin converting enzyme inhibitors; ARB, angiotensin receptor blockers; NSAIDs, non-steroidal anti-inflammatory drugs. OAD, oral anti-diabetic drugs.

### Main Outcomes

We investigated the development of the following conditions: CKD, end-stage renal disease (ESRD) defined as patients receiving dialysis, sight-threatening retinopathy defined as patients with at least two outpatient visits or one admission for retinopathy requiring surgery or receiving laser photocoagulation within 90 days of retinopathy diagnosis, or with visual loss, or receiving anti-vascular endothelial growth factor injection (ranibizumab, bevacizumab, or aflibercept); leg amputation defined by the ICD coding in at least one hospitalization. The incidence rates of CKD, ESRD, sight-threatening retinopathy, and leg amputation were calculated and compared between the comparison subgroups within each study cohort.

### Statistical Analysis

Propensity score matching was used to optimize comparability between the comparison subgroups within each study cohort (17). The propensity score for every patient was estimated using non-parsimonious multivariable logistic regression. Approximately 20 clinically related variables were used in the analysis as controlling variables (**Tables 1**– **3**). A standardized mean difference (SMD) algorithm was utilized to construct matching pairs under the assumption that a standardized mean difference of 0.05 or less indicated a negligible difference.

The incidence rates for each outcome were measured by the number of cases per 1,000 person-years. The person-years were calculated as the time from the index date to the date of the event, death, or the end of follow-up (December 31st, 2015), whichever came first. Crude and multivariate-adjusted Cox proportional hazard models were employed to compare the risk of outcomes between the study and comparison groups. The proportional hazards assumption was not violated by comparing estimated log-log survival curves for all time independent covariates. The results were presented as hazard ratios (HRs) and 95% confidence intervals (CIs) for study versus comparison groups. Because the competing risks of death might confound the estimates of risks for our investigated outcomes, we applied the Fine and Gray’s sub-distribution hazard model for adjustment. To assess risk for each investigated outcome, we censored patients on the date of death, the date of respective outcomes, or end of follow-up on 31 December 2015, whichever occurred first. A two-tailed *P* value less than 0.05 was considered significant. SAS v9.4 (SAS Institute, Inc., Cary, NC, USA) was used for the analysis.

## Results

### Study Population

In the diabetes cohort, after propensity score matching, 28943 pairs of matched patients were selected (**Table 1**). The mean follow-up time was 3.69 years for diabetes persons with subsequent hypertension and 3.59 years for persons without subsequent hypertension. In the hypertension cohort, 89102 pairs of matched patients were selected (**Table 2**). The mean follow-up time was 4.28 years for hypertension persons with subsequent diabetes and 4.26 years for persons without subsequent diabetes. In the cohort of coexisting diabetes and hypertension, 145294 pairs of matched patients were selected (**Table 3**). The mean follow-up time was 3.79 years for persons with previous diabetes and 4.07 years for persons with previous hypertension.

### Main Outcomes

In people with diabetes, those with subsequent hypertension had substantially higher risks of CKD (aHR=2.77, 95% CI 2.61-2.94) and ESRD (aHR=42.38, 95% CI 22.62-79.4) compared to those without hypertension (**Table 4**). In patients with hypertension, those with subsequent diabetes showed prominently higher risks of sight-threatening retinopathy (aHR=3.41, 95% CI 3.14-3.71) and leg amputation (aHR=6.27, 95% CI 4.72-8.31) than those without diabetes (**Table 4**). In patients with coexisting diabetes and hypertension, those with a previous history of hypertension showed a significantly lower risk of ESRD than those with previous diabetes (aHR=0.72); patients with a history of hypertension exhibited higher risks of CKD, sight-threatening retinopathy, and leg amputation than those with a history of diabetes (aHR: 1.04, 1.51, and 1.19, respectively **Table 4**).

**Table 4:** HRs and 95% CIs for the outcomes of CKD, ESRD, retinopathy, and amputation.

Outcome	Diabetes persons						cHR	(95% CI)	p-value	aHR^a^	(95% CI)	p-value
	Without subsequent hypertension (n = 28943)			With subsequent hypertension(n = 28943)								
	n	PY	IR	n	PY	IR						
CKD	1438	214511	6.7	3837	207142	18.52	2.78	(2.62, 2.96)	<0.001	2.77	(2.61, 2.94)	<0.001
ESRD	10	219012	0.05	407	220305	1.85	40.8	(21.8, 76.41)	<0.001	42.38	(22.62, 79.4)	<0.001
Sight-threatening retinopathy	483	216802	2.23	998	216496	4.61	2.07	(1.85, 2.3)	<0.001	2.07	(1.85, 2.3)	<0.001
Leg amputation	23	218983	0.11	111	221107	0.5	4.78	(3.05, 7.5)	<0.001	4.74	(3.02, 7.43)	<0.001
**Outcome**	**Hypertension persons**						**cHR**	**(95% CI)**	**p-value**	**aHR**	**(95% CI)**	**p-value**
	**Without subsequent diabetes (n=89102)**			**With subsequent diabetes (n=89102)**								
	**n**	**PY**	**IR**	**n**	**PY**	**IR**						
CKD	9179	781780	11.74	15028	751578	20	1.73	(1.68, 1.77)	<0.001	1.73	(1.68, 1.77)	<0.001
ESRD	332	819586	0.41	890	812346	1.1	2.74	(2.42, 3.11)	<0.001	2.76	(2.43, 3.13)	<0.001
Sight-threatening retinopathy	722	816799	0.88	2380	799469	2.98	3.37	(3.1, 3.66)	<0.001	3.41	(3.14, 3.71)	<0.001
Leg amputation	56	820498	0.07	348	813669	0.43	6.3	(4.75, 8.35)	<0.001	6.27	(4.72, 8.31)	<0.001
**Outcome**	**Coexisted diabetes and hypertension persons**						**cHR**	**(95% CI)**	**p-value**	**aHR**	**(95% CI)**	**p-value**
	**Previous diabetes history (n=145294)**			**Previous hypertension history (n=145294)**								
	**n**	**PY**	**IR**	**n**	**PY**	**IR**						
CKD	17591	1338198	13.15	20497	1309776	15.65	1.2	(1.17, 1.22)	<0.001	1.04	(1.02, 1.07)	<0.001
ESRD	1382	1414080	0.98	1221	1395614	0.87	0.91	(0.84, 0.98)	0.01	0.72	(0.66, 0.79)	<0.001
Sight-threatening retinopathy	3242	1397556	2.32	4380	1369825	3.2	1.38	(1.31, 1.44)	<0.001	1.51	(1.43, 1.58)	<0.001
Leg amputation	413	1417057	0.29	520	1397342	0.37	1.28	(1.13, 1.46)	<0.001	1.19	(1.03, 1.38)	0.02

CKD, chronic kidney disease; ESRD, end-stage renal disease; PY: person-years; IR: incidence rate, per 1000 person-years; cHR, crude hazard ratio; aHR: adjusted hazard ratio; CI, confidence interval.

aHR^a^: multivariable analysis including sex, age, obesity, smoking status, comorbidities, CCI, DCSI scores, medications, number of oral antidiabetic drugs, and diabetes or hypertension duration.

In brief, diabetes seemed to be an important risk factor for developing ESRD, sight-threatening retinopathy, and leg amputation; and hypertension was also an overlooked worsening factor for CKD and ESRD as shown in this study.

## Discussion

Our study demonstrated that (1). Among patients with diabetes, those with subsequent hypertension showed higher risks of CKD, ESRD, sight-threatening retinopathy, and leg amputation than those without subsequent hypertension. (2). Among patients with hypertension, those with subsequent diabetes demonstrated higher risks of CKD, ESRD, sight-threatening retinopathy, and leg amputation than those without subsequent diabetes. (3). Among patients with coexisting diabetes and hypertension, those with previous hypertension showed increased risks of CKD, retinopathy, and leg amputation, while those with a previous history of diabetes exhibited a higher risk of ESRD.

Approximately 10-15% of the population (18) and nearly 700 million people worldwide have CKD (5). CKD can increase the risk of cardiovascular disease and significantly shorten life expectancy (18). In 2019, approximately 1.4 million people died from CKD (4). CKD was the 12^th^ global leading cause of death in 2017 (19). Diabetes is the main risk factor for CKD (18, 19), and estimates suggest that about 50% of persons with type 2 diabetes will develop CKD (20). A cross-sectional study in Korea revealed that patients with diabetes showed a higher risk of CKD than patients with hypertension (21). Our study also demonstrated that patients with hypertension and subsequent diabetes showed a higher risk of CKD. Hyperglycemia may produce reactive oxygen species (ROS). ROS plays a key role in mesangial matrix expansion, tubule-interstitial fibrosis, podocyte loss, and CKD development (20). Several studies have revealed that intensive glucose control in persons with diabetes can reduce the risk of incident CKD, especially in reducing proteinuria (20). However, the best way to reduce the risk of incident CKD may be to prevent the occurrence of diabetes. Patients with hypertension should reduce the intake of sugar-sweetened beverages, control obesity, and increase physical activity to reduce the incidence of diabetes and mitigate CKD risk.

In 2010, approximately 31.1% of adults (1.39 billion) worldwide had hypertension (22). Hypertension is an important risk factor for CKD development and progression (18, 19). CKD can multiply the risk of cardiovascular death in patients with diabetes and hypertension (19). The study by the Global Burden of Metabolic Risk Factors for Chronic Diseases Collaboration reported that high blood pressure accounts for 45-46% of CKD deaths (23). Our study showed that patients with diabetes and subsequent hypertension and patients with coexisting diabetes and hypertension with a previous history of hypertension exhibited a higher risk of incident CKD. Shear stress in hypertension may induce endothelial dysfunction, impair renal autoregulation, change renal blood flow, activate the renin-angiotensin-aldosterone system (RAAS), and result in CKD (20). A meta-analysis revealed that intensive blood pressure lowering strategies could significantly reduce the risk of albuminuria but with no significant lowering of ESRD risk (24). Patients with diabetes should avoid excessive dietary sodium, control obesity, engage in physical activity, and reduce alcohol consumption to mitigate hypertension development and attenuate CKD risk (25).

ESRD is a condition with GFR < 15 ml/min/1.73 m^2^ or the need for dialysis or renal transplantation. Approximately 45% of patients with ESRD had type 2 diabetes in Taiwan (26). Up to 80% of ESRD was caused by diabetes, hypertension, or coexisting diabetes and hypertension (6). Both diabetes and hypertension are important prognostic factors for the progression of CKD to ESRD (20). A cohort study showed that the presence of diabetes could worsen patients with CKD to ESRD (27). The Multiple Risk Factor Intervention Trial established a consistent relationship between increased blood pressure and higher ESRD risk with the independence of relevant variables (28). Our study revealed that persons with diabetes and subsequent hypertension and patients with hypertension and subsequent diabetes showed a significantly higher risk of ESRD; especially persons with diabetes and subsequent hypertension had a very high adjusted HR [42.38(22.62-79.4)] for ESRD compared to persons without subsequent hypertension. Adding hypertension to persons with diabetes significantly increased the risk of ESRD. However, in patients with coexisting diabetes and hypertension, a previous history of diabetes seemed to have a higher impact on the risk of ESRD than a previous history of hypertension. This finding is consistent with previous reports that patients with a longer duration of diabetes showed a higher risk of ESRD (29). We must strive to mitigate the comorbidities of hypertension or diabetes to attenuate the progression of CKD to ESRD.

Approximately 35% of patients with type 2 diabetes have retinopathy. About 10% of patients with retinopathy have sight-threatening retinopathy (30) requiring close follow-up and aggressive treatments, such as vitrectomy, laser photocoagulation, or intravitreal anti-vascular endothelial growth factor injections to improve vision and avoid blindness. Taiwan Diabetes Atlas (2019) has reported that approximately 0.32% of persons with type 2 diabetes have sight-threatening retinopathy (31). The estimated global burden of retinopathy and sight-threatening retinopathy is 93 and 28 million individuals, respectively (30). Hypertension may worsen the progression of retinopathy (9), and suboptimal glycemic control may increase the retinopathy risk by 10–40% (29). Our study showed that persons with hypertension and subsequent diabetes and patients with comorbid diabetes and hypertension with a previous history of diabetes exhibited higher risks of sight-threatening retinopathy. Diabetes seems to play a crucial role in the development of sight-threatening retinopathy. However, patients with diabetes and subsequent hypertension also showed a significantly higher risk of sight-threatening retinopathy. Thus, the impact of hypertension on the risk of sight-threatening retinopathy cannot be ignored.

Inadequate treatment of foot ulcers or infection raises the risk of leg amputation, resulting in worsened quality of life in patients, reduced work performance, and impaired self-esteem (29). People with diabetes are 7–30 times more likely to receive non-traumatic leg amputations than the general population, accounting for more than half of all amputations (29). According to the Taiwan Diabetes Atlas (2019) report, approximately 1.16% of patients with type 2 diabetes had a diabetic foot, and 20.5% of these patients eventually needed leg amputations (31). Our study demonstrated that persons with diabetes and subsequent hypertension and persons with hypertension and subsequent diabetes showed an increased risk of leg amputation. We should strive to prevent subsequent hypertension development in patients with diabetes and subsequent diabetes development in patients with hypertension to reduce the risk of leg amputation in the future.

There are some disadvantages to this study. First, this dataset lacks information on blood pressure, glucose, hemoglobin A1C, renal function, urine protein, and retinal photographs to diagnose hypertension, diabetes, CKD, and retinopathy. We used the ICD codes to diagnose these diseases with acceptable accuracy, but there could have been potential errors. Some patients with mild or moderate retinopathy and mild renal dysfunction may escape detection with this protocol. Due to a lack of information on blood pressure and glucose, we attempted to match the numbers of antihypertensive drugs and antidiabetic drugs to balance the severity and treatment of hypertension and diabetes. Second, this administrative database lacks information on alcohol intake, family history, and physical activity. We tried to include more important variables, such as sex, age, obesity, smoking status, comorbidity, diabetes complication scores, and medications; we performed propensity score matching to increase the comparability between study and control groups. However, the unmeasured and unknown confounding factors still influenced our results. Third, the patients in this nationwide population-based study were mainly from Taiwan, and the results may not apply to other ethnicities. Finally, this study is a retrospective cohort study with some unobserved and unknown biases, and prospective studies are warranted to confirm our results.

In conclusion, CKD, retinopathy, and leg amputation are largely preventable and treatable diseases (19). Our study demonstrated that persons with diabetes and subsequent hypertension and persons with hypertension and subsequent diabetes showed significantly higher risks of incident CKD, ESRD, sight-threatening retinopathy, and leg amputation. This was rarely reported by previous studies (21). The family, school, and society should continuously educate people to avoid unhealthy lifestyles. Multifactorial interventions are necessary to mitigate comorbid hypertension or diabetes (28) and reduce the risk of nephropathy, retinopathy, and amputation.

## Data Availability Statement

Publicly available datasets were analyzed in this study. This data can be found here: Data of this study are available from the National Health Insurance Research Database (NHIRD) published by Taiwan National Health Insurance (NHI) Administration. The data utilized in this study cannot be made available in the paper. Requests for data can be sent as a formal proposal to the NHIRD Office (https://dep.mohw.gov.tw/DOS/cp-2516-3591-113.html) or by email to stsung@mohw.gov.tw.

## Ethics Statement

The studies involving human participants were reviewed and approved by Research Ethics Committee of China Medical University and Hospital (CMUH109-109-REC2-031). Written informed consent for participation was not required for this study in accordance with the national legislation and the institutional requirements. Written informed consent was not obtained from the individual(s) for the publication of any potentially identifiable images or data included in this article.

## Author Contributions

F-SY, Y-HS, and C-MH participated in the study design. JC-CW, C-CH, and Y-HS participated in the study coordination and data collection. Y-HS and C-CH participated in the data analysis; all authors contributed to the interpretation of the results and the discussion. F-SY, JC-CW, and C-MH participated in manuscript writing; all authors participated in revising the manuscript. C-CH and C-MH are the guarantors of this work, and have full access to all the data in the study, and take responsibility for the integrity of the data and the accuracy of data analyses. All authors contributed to the article and approved the submitted version.

## Funding

This study is supported in part by the Taiwan Ministry of Health and Welfare Clinical Trial Center (MOHW109-TDU-B-212-114004), MOST Clinical Trial Consortium for Stroke (MOST 109-2321-B-039-002), China Medical University Hospital (DMR-110-222), Tseng-Lien Lin Foundation, Taichung, Taiwan, and Taipei Veterans General Hospital (V101C-156, V108C-172, V109C-189), Taiwan. These funding agencies had no role in the study design, data collection and analysis, decision to publish, or manuscript preparation.

## Conflict of Interest

The authors declare that the research was conducted in the absence of any commercial or financial relationships that could be construed as a potential conflict of interest.

## Publisher’s Note

All claims expressed in this article are solely those of the authors and do not necessarily represent those of their affiliated organizations, or those of the publisher, the editors and the reviewers. Any product that may be evaluated in this article, or claim that may be made by its manufacturer, is not guaranteed or endorsed by the publisher.

## Abbreviations

CKD, chronic kidney disease; ESRD, end-stage renal disease; CCI, Charlson Comorbidity Index; DCSI, Diabetes Complication Severity Index.
