# Supplementary material for: The Risk of Nephropathy, Retinopathy, and Leg Amputation in Patients With Diabetes and Hypertension: A Nationwide, Population-Based Retrospective Cohort Study
Source: Front Endocrinol (Lausanne). 2021 Nov 18;12:756189. doi: 10.3389/fendo.2021.756189 (PMC8637105; doi:10.3389/fendo.2021.756189)
Supplement: Supplementary file 1 [file Table_1.docx]

Supplementary Material

**Supplementary Table 1 |** Diseases and related ICD-9-CM, ICD-10-CM codes.

| **Disease** | **ICD-9-CM codes** | **ICD-10-CM codes** |
| --- | --- | --- |
| Type 2 diabetes | 250.xx | E11 |
| Hypertension | 401–405, A26 | I10, I11, I12, I13, I15, N26 |
| Type 1 diabetes | 250.1x | E10 |
| Chronic kidney disease | 403.01, 403.11, 403.91, 404.02, 404.03, 404.12, 404.13, 404.92, 404.93, V42.0, 790 | E10.2, E10.65, E11.2, E11.65, E13.2, I12, I13, N03, N08, E10.21, E11.21, N05, N06, N07, N14, N15.0, N15.8, N15.9, N16, N17.1, N17.2, N19, Z94.0 |
| Dialysis | V56.0, V56.8, V45.1 | Z49.31, Z49.32, Z99.2 |
| Retinopathy | 362.02, 362.07, 362.0 | H35.0, E08.311-E08.359, E09.311-E09.359, E11.311-E11.359, E13.311-E13.359 |
| Visual loss | 369 | H54 |
| Leg amputation | V49.71–V49.77 or V52.1 | 0Y6 |
| Heart failure | 398.91, 402.01, 402.11, 402.91, 404.01, 404.03, 404.11, 404.13, 404.91, 404.93, 428, 429.4 | I09.81, I11.0, I13.0, I13.2, I50, I97.0, I97.110, I97.111, I97.120, I97.121, I97.130, I97.131, I97.190, I97.191 |
| Hepatic failure | 570, 572.2, 572.4, 572.8 | K72.00, K72.01, K72.10, K72.11, K72.90, K76.2, K72.90, K72.91, K76.7, K76.81 |
| Overweight | 278.02, 783.1, V85.2 | R63.5 |
| Obesity | 278.00, 649.1, V77.8, V85.3 | E66.09, E66.1, E66.8, E66.9, Z13.89 |
| Severe obesity | 278.01, 649.2, V45.86, V85.4 | E66.01, E66.2 |
| Smoking status | 305.1, 649.0, V15.82 | F17.200, F17.201, F17.210, F17.220, F17.221, F17.290, F17.291, Z87.891 |
| Dyslipidemia | 272 | E71.30, E71.31, E71.32, E71.39, E75.21, E75.22, E75.23, E75.24, E75.25, E75.29, E75.3, E75.4, E75.5, E75.6, E77, E78.0, E78.1, E78.2, E78.3, E78.4, E78.5, E78.6, E78.70, E78.71, E78.72, E78.79, E78.8, E78.9 |
| Coronary artery disease | 410-414 | I20, I21, I22, I24, I25.1, I25.2, I25.3, I25.4, I25.5, I25.6, I25.7, I25.81, I25.82, I25.83, I25.84, I25.89, I25.9 |
| Stroke | 430-438 | G45.0, G45.1, G45.2, G45.3, G45.4, G45.8, G45.9, G46, I60, I61, I62, I63, I65, I66, I67.0, I67.1, I67.2, I67.3, I67.4, I67.5, I67.6, I67.7, I67.8, I67.9, I68, I69 |
| Atrial fibrillation | 427 | I45.0, I45.1, I45.2, I45.3, I45.4, I45.5, I45.6 |
| Chronic obstructive pulmonary disease | 491, 492, 496 | J41, J42, J44, J43, J44.9 |
| Liver cirrhosis | 571.5, 571.2, 571.6 | K70.2, K70.30, K70.31, K74.0, K74.1, K74.2, K74.60, K74.69, K74.3, K74.4, K74.5 |
| Peripheral arterial occlusion disease | 440.0, 440.20, 440.21, 440.22, 440.23, 440.24, 440.3, 440.4, 443.9, 443.81, 443.89 | I70.2, I70.92, I75.0, I73.9 |
| End-stage renal disease | V56.0, V56.8, V45.1 | Z49.31, Z49.32, Z99.2 |
| Sight-threatening retinopathy | 362.02, 362.07, 362.0; NHI codes: 86206B, 86207B, 86407B, 86408B, 60001C, 60002C, 60005C, 60006C, 60003C, 60004C | H35.0, E08.311-E08.359, E09.311-E09.359, E11.311-E11.359, E13.311-E13.359; 08943ZZ, 08BE3ZZ, 08BF3ZZ, 08QE, 08QF, 085E3ZZ, 08QE3ZZ |

**Supplementary Table 2 |** The study design for the three cohorts in this study

1. Diabetes cohort

- Diabetes patients-------subsequent hypertension diagnosis (index date) -----------------------🡪 follow-up
  - the date of the first hypertension diagnosis was defined as the index date
- Diabetes patients without subsequent hypertension ---------(assigned an index date) -----------🡪 follow-up
  - matched to the “Diabetes with subsequent hypertension” individuals, and the same index date was assigned to the matched pairs

1. Hypertension cohort

- Hypertension patients-------subsequent diabetes diagnosis (index date) ---------------------------🡪 follow-up
  - the date of the first diabetes diagnosis was defined as the index date
- Hypertension patients without subsequent diabetes ---------(assigned an index date) ------------🡪 follow-up
  - matched to the “hypertension with subsequent diabetes” individuals, and the same index date was assigned to the matched pairs

1. Comorbid cohort

- The date of the first dual diagnosis of diabetes and hypertension was defined as the index date ----🡪 follow-up
- This cohort consists of two subgroups
  - Those with previous diabetes diagnosis before the first dual diagnosis of diabetes and hypertension
  - Those with previous hypertension diagnosis before the first dual diagnosis of diabetes and hypertension
  - We followed these two subgroups starting from the defined index date, and then compared their related outcomes
